# Alpha Band Cortico-Muscular Coherence Occurs in Healthy Individuals during Mechanically-Induced Tremor

**DOI:** 10.1371/journal.pone.0115012

**Published:** 2014-12-16

**Authors:** Francesco Budini, Lara M. McManus, Marika Berchicci, Federica Menotti, Andrea Macaluso, Francesco Di Russo, Madeleine M. Lowery, Giuseppe De Vito

**Affiliations:** 1 Department of Movement, Human and Health Sciences, University of Rome Foro Italico, Rome, Italy; 2 School of Public Health, Physiotherapy and Population Science, University College Dublin, Dublin, Ireland; 3 School of Electrical, Electronic and Communications Engineering, University College Dublin, Dublin, Ireland; 4 Neuropsychology Unit, IRCCS Santa Lucia Foundation, Rome, Italy; Harvard Medical School/Massachusetts General Hospital, United States of America

## Abstract

The present work aimed at investigating the effects of mechanically amplified tremor on cortico-muscular coherence (CMC) in the alpha band. The study of CMC in this specific band is of particular interest because this coherence is usually absent in healthy individuals and it is an aberrant feature in patients affected by pathological tremors; understanding its mechanisms is therefore important. Thirteen healthy volunteers (23±4 years) performed elbow flexor sustained contractions both against a spring load and in isometric conditions at 20% of maximal voluntary isometric contraction (MVC). Spring stiffness was selected to induce instability in the stretch reflex servo loop. 64 EEG channels, surface EMG from the biceps brachii muscle and force were simultaneously recorded. Contractions against the spring resulted in greater fluctuations of the force signal and EMG amplitude compared to isometric conditions (p<.05). During isometric contractions CMC was systematically found in the beta band and sporadically observed in the alpha band. However, during the contractions against the spring load, CMC in the alpha band was observed in 12 out of 13 volunteers. Partial directed coherence (PDC) revealed an increased information flow in the EMG to EEG direction in the alpha band (p<.05). Therefore, coherence in the alpha band between the sensory-motor cortex and the biceps brachii muscle can be systematically induced in healthy individuals by mechanically amplifying tremor. The increased information flow in the EMG to EEG direction may reflect enhanced afferent activity from the muscle spindles. These results may contribute to the understanding of the presence of alpha band CMC in tremor related pathologies by suggesting that the origin of this phenomenon may not only be at cortical level but may also be affected by spinal circuit loops.

## Introduction

Cortico-muscular coherence is a measure of the degree to which signals recorded from the sensory-motor cortex and skeletal muscle exhibit systematic phase-relations in specific frequency bands [1]. Oscillations in the alpha (8–12 Hz) and beta (13–35 Hz) frequency bands are commonly observed in recordings from the primary motor cortex [2]. Similar oscillations can also be detected in the electromyogram (EMG) of forearm and intrinsic hand muscles during sustained contraction [2], [3]. Although both cortical and muscle recordings exhibit oscillatory activity within the alpha and beta bands, in healthy subjects significant corticomuscular coherence is usually only observed within the beta band [4], [5], [6], [7], [8], [9], despite both frequency ranges being effectively transmitted to the corticospinal tract from the motor cortex [10]. Studies in subjects with Parkinson's disease and essential tremor, however, have demonstrated significant coherence between cortical activity and peripheral EMG in the alpha band (8–12 Hz) and the frequency range of pathological tremor (4–6 Hz) [11], [12], [13]. Furthermore, a significant peak in corticomuscular coherence has been reported at 8–12 Hz when healthy individuals imitated Parkinsonian resting tremor at 3–6 Hz [14]. This may suggest that the oscillations of peripheral limbs may induce coherence between the electrical activity of the sensory-motor cortex and skeletal muscle in the alpha band, regardless of displacement frequency.

It is known that oscillations of amplitude comparable to what is seen in tremor-related pathologies can be mechanically induced in healthy individuals during voluntary contractions against compliant loads of appropriated stiffness [15], [16], [17], [18], [19], [20]. These oscillations, referred to as mechano-reflex oscillations [21] or stretch reflex instabilities [16], have been attributed to enhanced afferent activity from the muscle spindles [17]. In the present study, we investigated the effects of mechanically amplified muscle tremor on the coherence between the electrical activity of the sensory-motor cortex and EMG recorded from the biceps brachii muscle. We hypothesised that alpha band cortico-muscular coherence occurs during mechanically-induced tremor in healthy individuals.

## Methods

### Participants

Thirteen individuals (age 23±4 years, body mass 69±12 kg, stature 1.77±0.1 m, 10 male and 3 female) with no history of neurological disorders volunteered for the experiment. The study was approved by the Ethics Committee of the University of Rome La Sapienza and written informed consent was obtained from all volunteers. The participants were requested to attend the laboratory for a single experimental session.

### Recording Apparatus

#### Force

**Figure 1:**
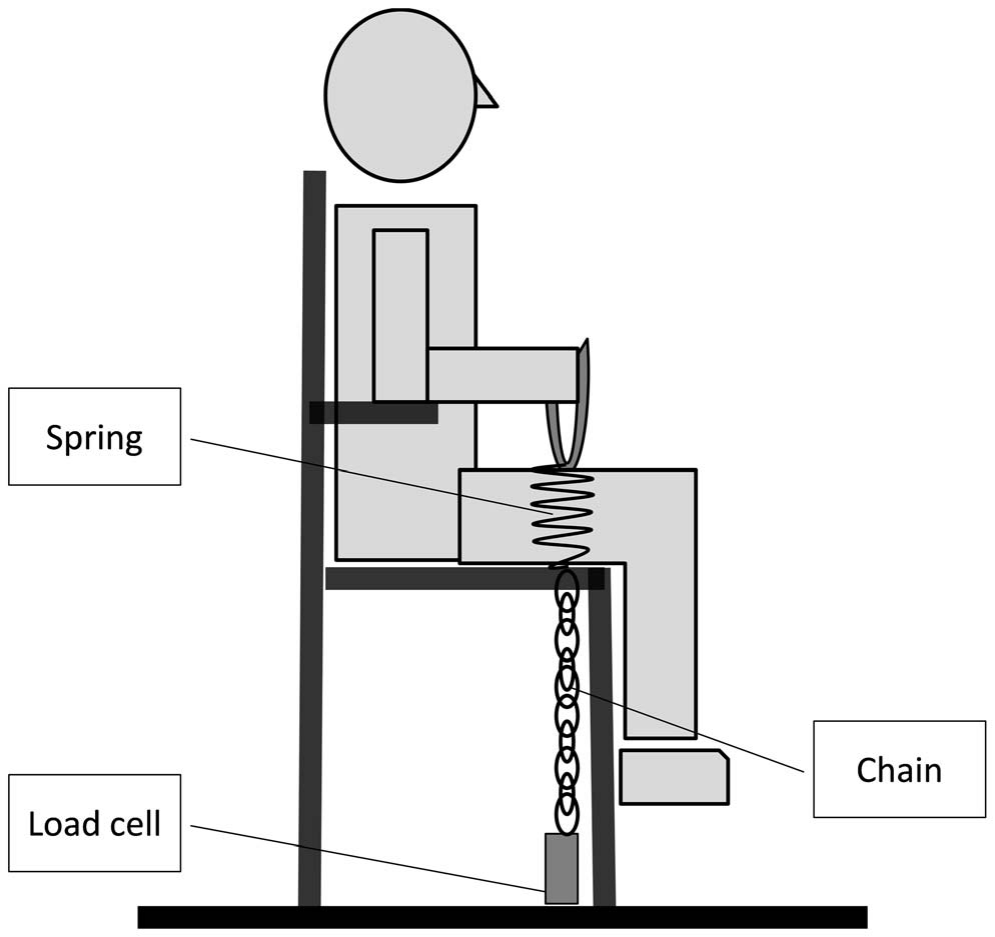
Schematic illustration of the experimental setup.

Participants were seated on a rigid custom-made chair with their trunk erect and fastened by an abdominal belt. The dominant arm was in line with the trunk and the elbow was flexed at 90° with the proximal third of the forearm resting neutral on the armchair and the distal two third unsupported and abducted by 40°. The participant held a handle connected to an inextensible chain, which in turn was linked to a piezoelectric force transducer (Kistler 9203, Winterthur, Switzerland) aligned with the direction of force application. Force output was assessed isometrically and against a compliant load. Isometric recordings were achieved by contracting against the handle through the inextensible chain. To achieve compliant recordings, a spring of appropriate stiffness was inserted between the handle and the inextensible chain, with the chain being shortened to maintain a 90° elbow angle (Fig. 1). The stiffness of the spring (3.22 N mm^−1^) was selected in order to generate stretch-induced tremor around the short latency reflex loop as demonstrated by Durbaba et al. [16]. The force signal was amplified (1K) (Kistler Charge Amplifier Type 5011, Winterthur, Switzerland), displayed on an oscilloscope in front of the participants (Tectronix TDS 220, Beaverton, USA), digitized with a sampling frequency of 2048 Hz and stored on a PC.

#### Electromyography

Surface electromyography (EMG) from the biceps brachii (BB) muscle of the dominant arm was detected using adhesive linear arrays of four electrodes (silver bars 5 mm long, 1 mm thick, 10 mm apart; LISiN, Torino, Italy) and recorded with an EMG amplifier (OT Bioelettronica, Torino, Italy). This electrode configuration allowed detection of three EMG signals in a single-differential mode from each array; the signal obtained from the middle electrode pair was used for analysis. After light skin abrasion and cleaning with alcohol the arrays were positioned, on the muscle belly following S.E.N.I.A.M. guidelines [22]. Conductive gel was used for each electrode to assure proper electrode-skin contact and was inserted with a syringe into the grooves of the adhesive electrode arrays. A ground electrode (Swaromed Universal, Nessler Medizintechnik GmbH, Innsbruck, Austria) was placed on the acromion process of the scapula. The EMG was amplified with a gain of 500, sampled at 2048 Hz and online band-pass filtered with cut-off frequencies of 3 and 500 Hz.

#### Electroencephalography

The EEG data were recorded using a BrainVision 64-channel system (Brain Products GmbH., Munich, Germany). Electrodes were placed according to the 10–10 system montage [23]. All scalp channels were referenced to the left mastoid. Horizontal eye movements were monitored with a bipolar recording from electrodes at the left and right outer canthi. Blinks and vertical eye movements were recorded with an electrode below the left eye, which was referenced to site Fp1. The EEG was digitized at 1000 Hz with an amplifier band-pass of 0.01–80 Hz together with a 50 Hz notch filter and stored for off-line averaging. Trials with artefacts (e.g. blinks or gross movements) were automatically excluded from averaging.

#### Data synchronization

A trigger was provided by the left click of a computer mouse sending an output signal to both EEG and EMG/force recording devices.

The synchronized force, BB EMG and trigger signals were imported into the AcqKnowledge software (Biopac Systems, inc. version 3.9.1). The EMG was low pass filtered at 400 Hz in order to prevent aliasing before being down sampled at 1000 Hz. Six selected channels for the EEG overlaying the sensory-motor cortex contralateral to the contracting arm (electrodes C3, C5, FC5, FC3, CP5 and CP3) and one central single differential signal of EMG were aligned through the trigger and saved as Matlab data files (7.8.0.347 R2009a) for the coherence analysis.

### Experimental Procedure

#### Maximal voluntary contraction

The MVC task consisted of rapidly increasing the force exerted by the right limb to a maximum. Participants were able to follow their performance on an oscilloscope (Tectronix TDS 220, Beaverton, USA) and were verbally encouraged to achieve a maximum and maintain it for at least 2–3 s before relaxing. The MVC was calculated as the peak value reached within any single force recording. Three attempts of MVC were performed, separated by 5 min, and the best of the 3 attempts was chosen as MVC. Further attempts were requested if the MVC of the last trial exceeded the previous one by at least 10% [24].

#### Submaximal contractions

Once the MVC value was determined, participants were asked to complete, in a random order, three isometric and three compliant sustained 40 s duration contractions at 20% MVC. Three-minute rest was allowed between efforts. During the contractions, the participants were provided with visual feedback of their performance and were instructed to maintain the force as close as possible to the visual force target represented by a horizontal cursor placed on the oscilloscope.

### Data analysis

Data from the three (isometric or compliant) submaximal contractions at 20% MVC were concatenated into single files of 120 s for analysis. The first 3 s of data were discarded to avoid transient phenomena.

The standard deviation of the filtered (1–40 Hz) force signal was calculated to estimate the amplitude of the oscillations in force. A frequency analysis of the force signal was also performed and the maximum value of the power spectral density and the integral within the alpha band were estimated.

Calculation of coherence between EEG and EMG was performed using Neurospec-software for Matlab (Neurospec, version 2.0, 2008, for a theoretical framework see [25]). Coherence was expressed as the maximal value of coherence within each frequency band [26]. Average coherence across six electrodes for each frequency band was selected for the analysis. To examine the direction of information flow between the cortical signals and the contralateral muscle partial directed coherence (PDC) was also calculated.

To assess Granger causality within a process of *m* different time series [(*x*_1_(*t*), *x*_2_(*t*),…*x*_m_(*t*))^T^], the time series are first detrended to remove the mean value or linear trend so that they have approximately constant mean and variance. The time series are then modelled through a vector autoregressive (VAR) model of the form:
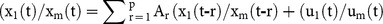
where [(u_1_(t), …u_m_(t))^T^] are uncorrelated Gaussian white noise processes representing the model residuals, with covariance matrix Σ. The VAR model's order *p* represents the maximum temporal delay in the causal link between the modelled time series. The VAR coefficients *A_r_* are *m*×*m* matrices, with each entry *A_r_(ij)* corresponding to the linear effect of *x_j_*'s past value *x_j_(t*−*r)* on *x_i_*'s present. The Fourier Transform is then performed on the matrix of VAR coefficients to produce a frequency-domain account of the VAR model. The PDC from time series *x*_j_ to *x*_i_ is defined as:
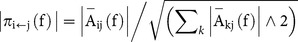
where *Ā(f)* is the difference between the identity matrix and *A(f)* (i.e. *Ā(f)*  = I− *A(f)*). The PDC π_i←j_(*f*) is zero for all frequencies only if *A_r_(ij)*  = 0 for all 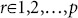.

An extended version of PDC is used in the analysis, information partial directed coherence (iPDC) [27], which uses information-theory formulations to explicitly define the relationship between information flow and PDC. This method provides an absolute signal scale invariant measure of direct connectivity strength between two structures as opposed to original and generalized PDC that provide only relative coupling assessments.

The alpha band corticomuscular coherence was analyzed for 3 EEG data channels (FC3, C5, CP5). The model order *p* must be set before VAR modeling of the time series takes place, *p* represents the delay of information transfer between the recorded brain or muscle regions. A model order of 200 was chosen for analysis, which equates to a time lag of 200 ms. The null hypothesis of significant PDC was tested via its computed asymptotic statistical properties [27]. Directions of the interrelations between the left EEG and right EMG recorded were determined, with PDC values were calculated for windows of 8 seconds with a time step of 1 second. The sum of all significant partial directed coherences was calculated per subject for both isometric and spring conditions, for each direction of information flow, in the alpha and beta bands. The average value and standard deviation of the summed PDC values were determined across all subjects per condition.

### Statistics

Statistical comparison of the coherence between bands (alpha and beta) and contraction types (isometric and spring) were carried out using two-way analysis of variance (ANOVA) for repeated measures, to look at the effect of band (alpha vs. beta), condition (isometric vs. spring), and interaction between factors. Paired student t-tests were used to compare EMG RMS and force displacement between the two contraction conditions. For partial directed coherence, a paired t-test was performed between the isometric and spring conditions for both the EEG to EMG and EMG to EEG directions.

The maximum value and integral of the power spectrum of the force signals showed a skewed distribution, for this comparison a Wilcoxon rank test was, therefore, adopted.

An alpha level of p<.05 was accepted as indicated a statistically significant difference between conditions.

## Results

### Effects of spring on force and EMG

**Figure 2:**
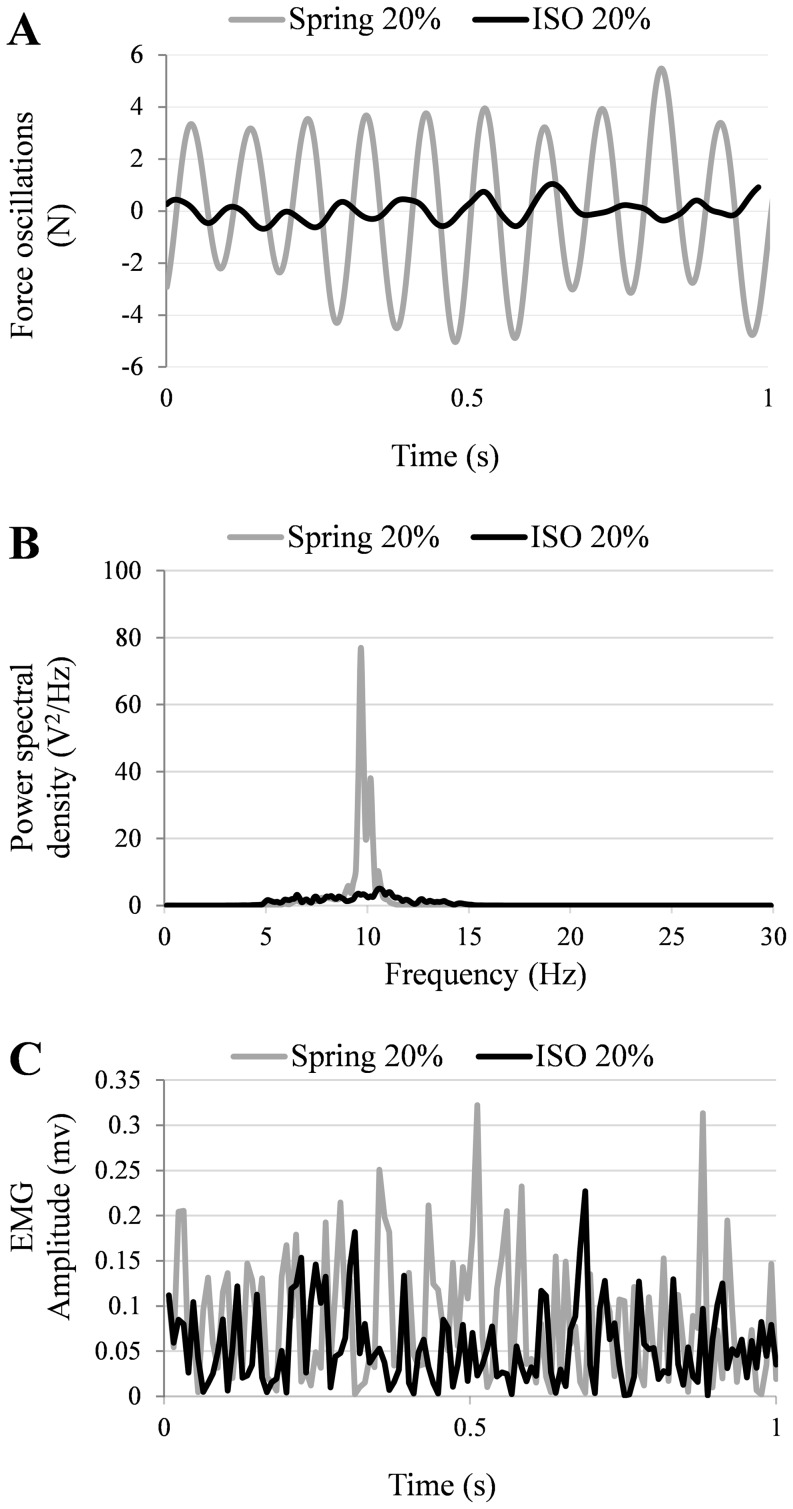
Comparison of force oscillation (A), force power spectral density (B) and EMG amplitude (C) between isometric (black lines) vs. spring (grey lines) contractions in a representative volunteer. For plot A, mean force values were subtracted from the force signals and force data were low pass filtered (20 Hz).

Fig. 2A shows 1 s segment of the force recordings from a representative participant during both isometric (ISO) and compliant (Spring) contractions; the corresponding rectified muscle electrical activity is plotted in panel 2C. The power spectral density of the force for the entire 120 s contraction is plotted in panel 2B. Visual inspection of the force signals and corresponding power spectra shows a similar frequency of oscillation (∼10 Hz) but a larger displacement during the contractions against the spring load. Similarly, for this subject, a noticeable difference in the amplitude of the EMG between the two contraction conditions is evident, with the contractions against the spring being accompanied by higher muscle electrical activity (Fig. 2C).

**Figure 3:**
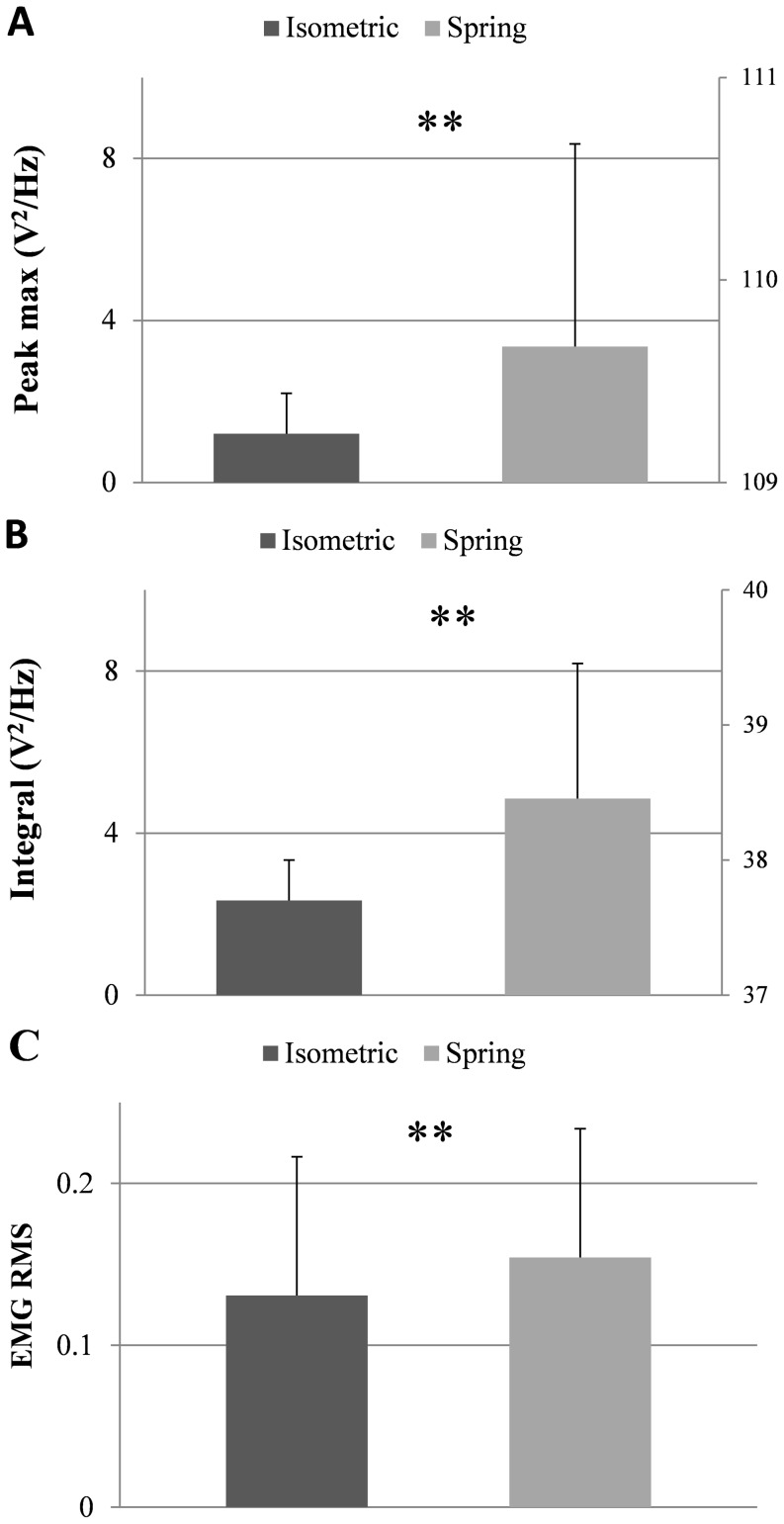
Group average of the maximum value (A) and integral beneath the peak (B) of the force power spectral density during isometric (black bars) and contractions against the spring (grey bars), double y axes were used in these plots. (C) EMG RMS.**p<.01 (paired t-test).

The results reported for a representative subject are consistent with the average data of the group. As shown in Fig. 3, the maximum value and integral of force power spectral density within the alpha band during the sustained sub-maximal efforts were higher in the contractions with the spring than in the isometric conditions (p = 0.003, Z = −2.934 for both; Fig. 3A and 3B). Similarly, the RMS EMG was higher during the contractions against the spring (p = 0.009) compared to the isometric conditions (Fig. 3C).

### Coherence analysis

**Figure 4:**
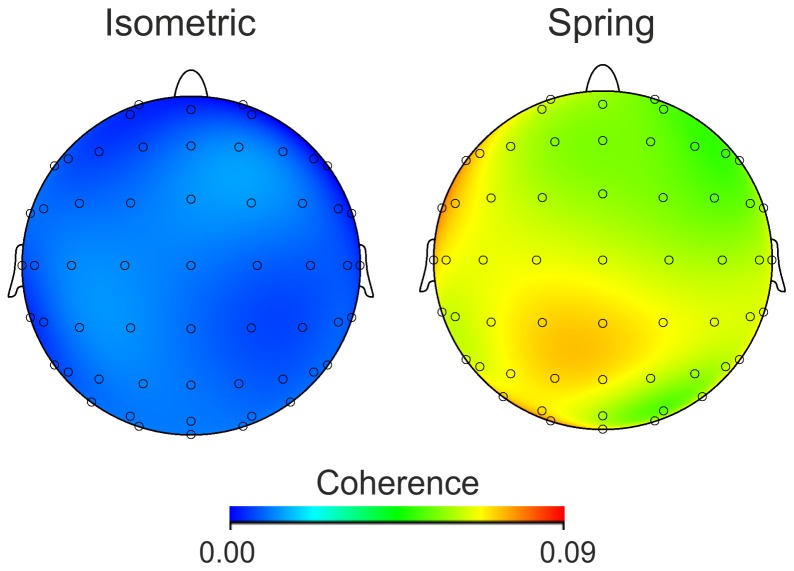
Grand-average alpha band CMC across all participants in the two experimental conditions.

**Figure 5:**
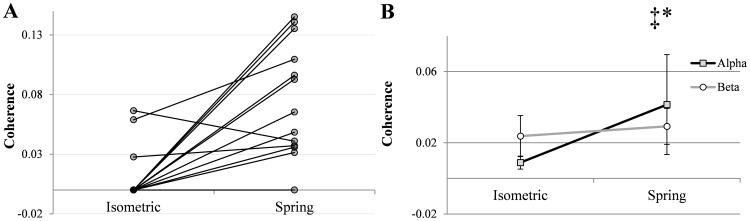
Coherence in the alpha band during isometric contractions and contractions against the spring. 5A) Dots indicate the cortico-muscular coherence peak value for each volunteer, obtained from the EMG and the EEG sensor pair with the highest reported value of alpha band coherence during contractions against the spring. In this graph, when coherence was below significance level, coherence values were reported equal to “0”. 5B) Group average maximal peak coherence across 6 EEG channels in the alpha and beta bands during isometric contractions and contractions against the spring. *Condition effect p<.05; ‡ Interaction effect p<.05 (Two way ANOVA for repeated measures)

During isometric contractions, significant EEG-EMG coherence between at least one EEG sensor and the EMG was observed in the beta band frequency range in 10 subjects. Further significant coherence was noted in the alpha band in 3 subjects. During compliant contractions, the total number of subjects exhibiting cortico-muscular coherence between at least one EEG sensor and the EMG in the alpha band increased from 3 to 12. Fig. 4 shows group average CMC in the alpha band during both isometric and spring contractions. The effect of the spring was particularly marked in 7 individuals, as shown in Fig. 5A.

ANOVA revealed a significant effect of condition (p = 0.033, F = 5.924), with the peak mean coherence in the alpha band showing an average increase from 0.022±0.019 in the isometric condition to 0.106±0.11 during contraction against the spring (Fig. 5B). No significant change in beta band coherence was observed, whilst the effect of interaction band/condition was significant (p = 0.038 F = 5.532; Fig. 5B).

**Figure 6:**
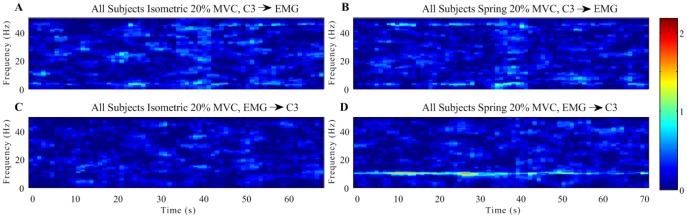
Partial directed coherence grand-averages across all the subjects shows the detection of significant information flow in the EEG to EMG direction (A and B) and the EMG to EEG direction (C and D) over time for isometric (A and C) and spring (B and C) conditions.

**Figure 7:**
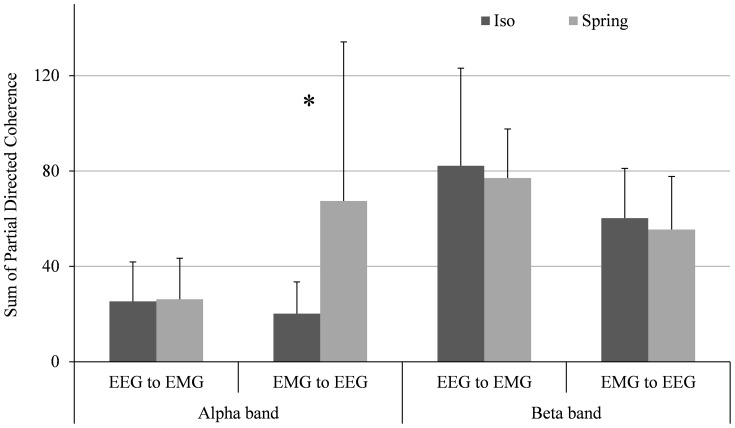
Bar plot comparing average PDC values and standard deviation in the alpha and beta frequency bands for both isometric and compliant sustained contractions. * p<.05 (paired t-test)

Significant PDC values were calculated for each subject in 1 second time steps and summed to show the collective PDC across all subjects. Causal influences above threshold were detected in the EEG to EMG (Fig. 6A and B) and EMG to EEG direction (Fig. 6C and D), for both isometric and spring conditions. A significant increase in alpha band coherence was observed from EMG to EEG with the addition of the spring (*p* = 0.013). No significant increase was identified for the beta band (Fig. 7).

## Discussion

In this study, we have shown that coherence in the alpha band between the sensory-motor cortex and the biceps brachii muscle can be systematically induced in healthy individuals by mechanically amplifying tremor. The increased information flow in the EMG to EEG direction suggests enhanced afferent muscle spindle activity in response to stimulation of the stretch reflex servo loop. This result may have implications for the understanding of alpha band corticomuscular coherence in tremor-related pathologies by suggesting that not only abnormalities in cortical inhibition [13], but also spindle-induced spinal loop reflex activity could be responsible for the synchronization between cortical and muscular activity in the 8-12 Hz frequency band [21].

The greater amplitude of the force oscillations during compliant contractions is consistent with the findings of others [16], [18], [20] and has been attributed to the contribution of the stretch reflex, which enhances the oscillations [28], [17] to the extent that it overwhelms neural mechanisms that would minimize the limb displacement under isometric, isotonic or postural contraction. The higher EMG amplitude during the compliant contractions, with respect to the isometric contractions, likely reflects enhanced motoneuron activity due to afferent input from the muscle spindles, which results in a summation of background EMG and M reflexes [29]. This result is also in accordance with previous findings of Budini et al. [15], who observed that EMG activity decreased with decreasing tremor.

Significant peaks in the EEG-EMG coherence spectra were observed in the beta band frequency range during both the sustained isometric and compliant contractions, which is consistent with several previous studies [3], [4], [5], [6], [7], [8], [9]. Coherence in the alpha frequency band was observed in only a few participants during isometric contractions, also consistent with previous observations of Ushiyama et al. [30]. Sustained submaximal contractions performed against the spring load, however, showed a marked systematic effect on cortico-muscular coherence resulting in a clear, significant peak in the alpha band in 12 out of 13 participants, as shown in Fig. 5.

The absence of an increase of corticomuscular coherence in the beta band is in agreement with other studies [13], [14] showing that wide peripheral oscillations of a limb are predominantly related to alpha band corticomuscular coherence. These results are in contrast with Kilner et al. [7], who observed no occurrence of alpha band corticomuscular coherence during contractions of the first dorsal interosseous against a compliant load. However, this discrepancy may be explained by the limited amplitude of the mechanically induced oscillations in the first dorsal interosseous [16], which is attributed to the weakness of stretch reflexes in this muscle [31]. In the present study, the CMC may arise from coupling between muscle spindle activity and the somatosensory cortex which is known to receive powerful input from muscle receptors [32] and also to modulate alpha oscillations [33].

The implication of muscle spindle afferent activity in the onset of alpha CMC can also be hypothesised on the base of its effects on Renshaw cells. Recurrent inhibition via Renshaw cells may contribute to decorrelate corticomuscular alpha band coherence [34]. Renshaw cells activity can be depressed by low-threshold afferent neurons such as the secondary muscle spindle afferents as demonstrated both on decerebrated cats [35] and through computer modelling [36] but are excited by muscle spindles primary afferents [37]. Therefore, an enhanced muscle spindle afferent activity might have provoked the onset of alpha band corticomuscular coherence by influencing Renshaw cells action which, in an otherwise normal functioning state, would have decorrelated cortical and muscular signals.

A similar phenomenon may also occur during pathological tremors where the rhythmic oscillations of the body segment are likely accompanied by continuous activations of muscle spindles with a related augmented afferent contribution. It could, therefore, be suggested that the alpha band cortico-muscular coherence observed in Parkinson patients could be attributed not only to pathological synchronization of neuronal activity as suggested by Timmerman et al. [13], but also to a mechanically enhanced spinal loop reflex activity.

The relevance of afferent pathways on the onset of corticomuscular coherence is consistent with other recent studies [38], [39]. The increase in alpha band partial directed coherence from EMG to EEG (Fig. 7) supports the hypothesis that the spring-induced instability around the stretch reflex enhanced muscle spindle afferent activity. The lack of a significant increase in alpha band PDC from EEG to EMG suggests that another neural circuit may be acting in this direction to filter ∼10 Hz inputs to the motoneuron pool. Williams et al. [40] hypothesized that excitatory spinal circuit interneurons also participate in reducing oscillations by phase-inverting inputs to motoneurons. The convergence of antiphase activity from spinal motoneurons with descending oscillations from the cortex and subcortical centers results in cancellation, diminishing the amplitude of oscillations transmitted to the periphery [41].

In conclusion, it was demonstrated that cortico-muscular coherence in the alpha band can be systematically induced by modifying the dynamics of an oscillating system through the use of a spring of appropriated stiffness.

The generation of alpha band corticomuscular coherence may arise partially through the reflection of muscle activity in the contralateral sensorimotor cortex via proprioceptive afferents, and is not solely caused by oscillatory activity transmitted from the cortex to muscle via the corticospinal tract [41]. Significant causal influences were transiently detected from the EEG to the contralateral EMG, indicating that the motor cortex does play a role in tremor generation (Fig. 6B). However, in this study, afferent feedback from the muscles to the cortex appears to play a more dominant role, with a significant increase in alpha band partial directed coherence from EMG to EEG observed during the contractions against a spring. The lack of a similar significant increase in the EEG to EMG direction would support the suggestion that multiple neural systems exist that limit the motoneuron pool from synchronizing with the 10 Hz oscillations present in the cortical descending command.

Future animal studies may clarify the role of the muscle spindles in the synchronization between cortical and muscular activity in the alpha band in individuals with pathological tremor.
